# *Bifidobacterium adolescentis* induces Decorin^+^ macrophages via TLR2 to suppress colorectal carcinogenesis

**DOI:** 10.1186/s13046-023-02746-6

**Published:** 2023-07-18

**Authors:** Yifeng Lin, Lina Fan, Yadong Qi, Chaochao Xu, Dingjiacheng Jia, Yao Jiang, Shujie Chen, Liangjing Wang

**Affiliations:** 1 Department of Gastroenterology, Second Affiliated Hospital of Zhejiang University School of Medicine, Hangzhou, Zhejiang Province 310009 China; 2 Institute of Gastroenterology, Zhejiang University, Hangzhou, China; 3 Department of Gastroenterology, School of Medicine, Sir Run Run Shaw Hospital, Zhejiang University, Hangzhou, Zhejiang Province 310003 China; 4 Cancer Center, Zhejiang University, Hangzhou, Zhejiang China; 5 Research Center of Prevention and Treatment of Senescent Disease, School of Medicine, Zhejiang University, Hangzhou, China

**Keywords:** Colorectal cancer, *B.adolescentis*, Macrophages, Decorin, TLR2

## Abstract

**Background:**

The interplay between gut microbiota and tumor microenvironment (TME) in the pathogenesis of colorectal cancer (CRC) is largely unknown. Here, we elucidated the functional role of *B. adolescentis* and its possible mechanism on the manipulation of Decorin^+^ macrophages in colorectal cancer.

**Methods:**

The relative abundance of *B. adolescentis* in tumor or para-tumor tissue of CRC patients was analyzed. The role of *B. adolescentis* was explored in the CRC animal models. The single cell-RNA sequencing (scRNA-seq) was used to investigate the myeloid cells subsets in TME. The expression level of TLR2/YAP axis and its downstream Decorin in macrophages were tested by Western blot and qRT-PCR. Knockdown of *Decorin* in Raw264.7 was performed to investigate the effect of Decorin^+^ macrophages on subcutaneous tumor formation. Multi-immunofluorescence assay examined the number of Decorin^+^ macrophages on the CRC tissue.

**Results:**

We found that the abundance of *B. adolescentis* was significantly reduced in tumor tissue of CRC patients. Supplementation with *B. adolescentis* suppressed AOM/DSS-induced tumorigenesis in mice. ScRNA-seq and animal experiment revealed that *B. adolescentis* increased Decorin^+^ macrophages. Mechanically, Decorin was activated by TLR2/YAP axis in macrophages. The abundance of *B. adolescentis* was correlated with the number of Decorin^+^ macrophages and the expression level of *TLR2* in tumor tissue of CRC patients.

**Conclusions:**

These results highlight that *B. adolescentis* induced Decorin^+^ macrophages and provide a novel therapeutic target for probiotic-based modulation of immune microenvironment in CRC.

**Supplementary Information:**

The online version contains supplementary material available at 10.1186/s13046-023-02746-6.

## Introduction

Colorectal cancer (CRC) ranks third in incidence and mortality globally [1]. Gut microbes are closely bound up with CRC via immune cells [2]. Accumulating evidence suggested that several special bacteria connected with the initiation and progression of CRC through immune pathway [3–5]. For instance, *Helicobacter hepaticus* induces T follicular helper cells and tertiary lymphoid structures to against CRC [6]. Among all the immune cells, macrophages are regarded as a critical part interacting with the intestinal flora in CRC. The gut microbiota regulates monocyte-like macrophages accumulation in a chemokine dependent manner and mediates an inflammatory response to facilitate colitis-associated tumorigenesis [7]; Tryptophan metabolites derived from microbes activate the aryl hydrocarbon receptor of tumor-associated macrophages to suppress anti-tumor immunity [8]. Accordingly, utilizing the flora to regulate macrophages is a potential therapeutic strategy in CRC [9].

*Bifidobacterium* plays a critical role in immune maturity and regulation [10–12]. Studies suggested that *Bifidobacterium* was associated with enhanced efficacy of checkpoint blockade immunotherapy [13, 14]. For example, cocktail of *Bifidobacterium* (*B. breve* and *B.longum)* promotes anti-tumor immunity and improves anti-PD-L1 efficacy [15]. Intratumoral accumulation of *Bifidobacterium* cocktail (*B. bifidum, B. longum, B. lactis and B.breve*) facilitates CD47-based immunotherapy via STING signaling [16]. *Bifidobacterium pseudolongum* enhances the anti-CTLA-4 therapy response by producing the metabolite inosine [17]. Most of these studies on cancer focus on *Bifidobacterium* cocktail and checkpoint blockade immunotherapy. However, the potential role of *B.adolescentis* on initiation and progression of CRC remains poorly understand. Our previous work indicated that *B.adolescentis* ameliorated chronic colitis by regulating immune response and gut microbiota remodeling [18].

In this study, we observed that supplementation with *B.adolescentis* suppressed colorectal tumorigenesis. Mechanically, we uncovered that *B. adolescentis* induced Decorin^+^ macrophages infiltration in TME and Decorin in macrophages was a crucial molecule mediating the anti-tumor effect of *B.adolescentis*. In addition, *B. adolescentis* increased Decorin^+^ macrophages via a TLR2/YAP-dependent signaling. Our results highlighted the protective role of *B.adolescentis* on CRC, which provided a new therapeutic strategy based on probiotics modulation of tumor immune microenvironment.

## Methods

### Cell culture

Human colorectal cancer cell line (HCT116), mouse colorectal cancer cell line (CT26), human macrophage cell line (THP-1) and mouse macrophage cell line (Raw264.7) were purchased from American Type Culture Collection (ATCC). HCT116 cultured in Maccoy 5A (Genom, China) supplemented with 10% FBS (Sijiqing, China) at 37 °C in a humidified 5% CO_2_ atmosphere. CT26, THP-1 and Raw264.7 cultured in RPMI 1640 (GIBCO, China) supplemented with 10% FBS (Sijiqing, China) at 37 °C in a humidified 5% CO_2_ atmosphere.

### Bacterial strains and culture conditions

*B. adolescentis* was purchased from American Type Culture Collection (ATCC 15703). *B. adolescentis* grown in Reinforced Clostridium Medium (BD Difco, USA) was cultured under an atmosphere of 10% H_2_, 10% CO_2_, and 80% N_2_ in an AW500SG anaerobic workstation (ELECTROTEK) at 37 °C. The *E. coli* strain MG1655 (ATCC 700926) was cultured in Luria–Bertani medium overnight at 37 °C.

### DNA extraction and bacteria DNA quantification

Genomic DNAs extraction and purification from clinical or mice tissue were executed by QIAGEN DNA Mini Kit (Qiagen, Germany) according to the instructions. Quantitative real-time PCR was performed to assess the relative abundance of *B. adolescentis.* The abundance of *B. adolescentis* was calculated by -ΔCt method and the universal Eubacteria 16s was used as an internal reference gene. Primers used are listed in the Supplementary Table S1.

### Human specimens

A total of 65 paired fresh tumor and adjacent non-tumor tissue were obtained from patients with primary CRC who had not received preoperative anti-tumor and antibiotic treatment from Sir Run Run Shaw Hospital of Zhejiang University (Hangzhou, China). All samples were frozen in liquid nitrogen until use. Clinical Research Ethics Committee of the Sir Run Shaw Hospital, Zhejiang University School of Medicine approved the protocol. All aspects of the study were conducted in accordance with the principles of the Declaration of Helsinki.

### Carcinogen-induced cancer model

6-week-old male C57BL/6 wildtype mice were obtained from Shanghai SLAC Laboratory Animal, China. Before bacterial intragastric administration, as previous study reported, 2 mg/mL streptomycin (Cat. #MB1275, Meilunbio) solubilized in water was given to mice for 7 days to ensure the conformance of regular microbiota and facilitate *B. adolescentis* colonization. C57BL/6 mice were given one single intraperitoneal injection of carcinogen azoxymethane (AOM, Sigma) (10 mg/kg per mouse), and then were given 5 successive days of 2.5% dextran sodium sulfate (DSS, MP Biomedicals) in the drinking water, following by regular drinking water for 2 weeks. This cycle was then repeated for twice [19]. Two weeks later, mice in *B.adolescentis* and *E. coli* groups were administrated with 1 × 10^9^ CFU *B.adolescentis* or *E. coli* suspended in 200 μL sterile PBS every day, and the control group was administered with PBS. After about 3 months, mice were sacrificed and the colorectum was surgically excised for further analysis.

### Animal use and care

All animal studies were approved by the Institutional Animal Care and Use Committee (IACUC) of Zhejiang University (ZJU). All animal experiments strictly adhered to protocols, policies, and ethical guidelines formulated by our IACUC. BALB/c nude mice, BALB/c mice and C57BL/6 mice were purchased from Shanghai SLAC Laboratory Animal, China. All mice were maintained in ventilated cages with 12 h light/dark cycles, constant temperature and humidity, enriched water and ad libitum feeding under specific pathogen-free (SPF) conditions.

### Subcutaneous tumor models

Male BALB/c nude mice (4 weeks old) were reared in specific-pathogen-free (SPF) facilities. HCT116 cells were mixed with *B.adolescentis*, *E.coli* or PBS (MOI of 10:1) and macrophages (THP-1 or Raw264.7; cancer cells: macrophages = 1:1) with 50 μL Matrigel matrix (Cat. #354234,Corning Biocoat), and hypodermically injected into BALB/c nude mice (100 μL, 2 × 10^6^ cells/per mouse). For BALB/c mice (6-8 weeks old), CT26 cells were mixed with *B.adolescentis*, *E.coli* or PBS (MOI of 10:1) and Raw264.7 (CT26: Raw264.7 = 10:1) with 50 μL Matrigel matrix (Cat. #354234,Corning Biocoat), and hypodermically injected into BALB/c mice (100 μL, 2 × 10^6^ cells/per mouse). After 6-7 days inoculation, the tumor volume of the mice was examined regularly and calculated as follows: Volume = 0.54 × L × W^2^, where L is the longest diameter and W is the shortest diameter. For TLR2 inhibition, 3 mg/kg Cu-CPT22(Selleck Cat. #S8677) was injected intraperitoneally to mice every two days until sacrifice. For YAP inhibition, 50 mg/kg Verteporfin (MCE Cat. #CL 318952) was injected intraperitoneally to mice every day until sacrifice.

### Isolation of primary macrophages

For primary mouse macrophages BMDMs, bone marrow cells were harvested from 8-10 weeks old C57BL/6 mice as previously done [20]. 6 days later, BMDMs were confirmed by flow cytometry for CD11b and F4/80 and further used. For primary human macrophages, after isolating the CD14^+^ monocytes from PBMC using the MojoSort Human CD14 Selection Kit (Biolegend Cat No:480025) according to the manufacturer’s protocol, we cultured them in RPMI 1640 supplemented with 10% (v/v) FBS, penicillin/streptomycin, and 50 ng/mL M-CSF for 5 days to induce macrophages differentiation [21]. For polarization of M1 macrophages, BMDMs and THP-1 cells were treated by 100 ng/mL IFN-γ (Cat. #C746, Novoprotein) for 24 h as previous study reported [22], then the expression of aimed genes were examined by qRT-PCR.

### Cytotoxicity experiments

For cytotoxicity experiments, BMDMs were pretreated with *B.adolescentis*, *E. coli* or vehicle (PBS) for 24 h, and then co-cultured with HCT116 or CT26 (BMDMs: tumor cells = 1:1) for 24 h. The level of LDH was evaluated according to the manufacturer’s instructions [23].

### Isolation of colorectal lamina propria cells

Isolation of colorectal lamina propria cells was performed as previously done [20]. In brief, the posterior 1/3 of the colorectum without lymph and adipose tissue was cut into small pieces and washed in RPMI 1640 medium (GIBCO, China). The tissue was incubated in D-Hanks (Cat. #MA0039, Meilunbio) buffer supplemented with 1 mmol/L DTT (Cat. #MB30471, Meilunbio) and 5 mmol/L EDTA (Cat. #MB2514, Meilunbio) on a shaker (200 rpm) for 30 min at 37℃. And then the remaining colorectum was cut into 1 mm pieces and further digested in Hanks buffer (Cat. #MA0041, Meilunbio) supplemented with 1 mg/mL Type IV collagenase (Cat. #A005318, Sangon Biotech) for 30 min at 37℃ with 200 rpm shaking. After complete digestion, the cell suspension was passed through a 300-mesh filter and then centrifuged at 500 g for 5 min. The isolated colorectal lamina propria cells were further analyzed.

### Flow cytometry analysis

Cells were counted (5 × 10^6^ cells/per sample) and stained for 20 min at 4℃ Fixable viability stain 510 (Cat. #564406, 2 mg/mL, BD Biosciences) for live cell staining, then the surface molecules of the cells were stained with antibodies for 30 min at 4℃. The panel of antibodies designed to analyze of myeloid cells: Alexa Fluor 700-CD45 (Cat. #560510, Clone 30-F11, 1 mg/mL, BD Biosciences), BV421-F4/80 (Cat. #565411, Clone T45-2342, 1 mg/mL, BD Biosciences), BV605-CD11b (Cat. #557672, Clone M1/70, 1 mg/mL, BD Biosciences), PE-MHC-II (Cat. #557000, Clone M5/114, 1 mg/mL, BD Biosciences), PE-CY7-CD11c (Cat. #117317, Clone N418, 1 mg/mL, BioLegend) and APC-CD206 (Cat. #17-2061-82, Clone MR6F3, 2 mg/mL, eBioscience). Samples were analyzed using Flow Cytometer (BD Biosciences). Subsequent analysis was performed with FlowJo software (Tree Star Inc.).

### Cell culture in presence of *B.adolescentis*

BMDMs, THP-1 cells or Raw264.7 cells were seeded in 6-well plate at a density of 5 × 10^5^ cells per well and cultured in DMEM or RPMI  1640 medium with 10% (vol/vol) FBS overnight. BMDMs, THP-1 cells or Raw264.7 cells were co-cultured with *B.adolescentis* at a MOI = 100:1 for 24 h. Finally, the protein and RNA of BMDMs, THP-1 cells or Raw264.7 cells were extracted for analysis.

### RNA extraction and quantitative qRT-PCR

RNAs were extracted from macrophages or tissue samples using Trizol reagent (Invitrogen, USA), and total RNA were reversed by Evo M-MLV RT Kit (Accurate Biology, China) according to the manufacturer’s instructions. Quantitative RT-PCR analysis was performed in triplicate in ROCHE LightCycler480 System (Rotor gene 6000 Software, Sydney, Australia) with SYBR Premix Ex Taq (Takara, Japan). Relative abundance was demonstrated by -ΔCt method. Primers used are listed in the Supplementary Table S1.

### Genomics library and single cell RNA sequencing

Primary Sequencing data were demultiplexed and converted to FASTQ format by Illumina bcl2fastq software. Cell Ranger pipeline (version 3.1.0) was used to perform sample demultiplexing, barcode processing and single-cell 3, gene counting. Then scRNA-seq data were aligned to Ensemble genome GRCm38 reference genome. After filtering and UMI (universal molecular identifier) counting, a total of 18,661 single cell captured from 2 samples were captured using 10 × Genomics Chromium Single Cell 3’ Solution. The mean reads per cell was 49,151 with median UMI Counts per Cell of 5,790 after aggregation.

All bioinformatics analysis was performed using R version 3.4.0 (R Foundation, https://www.rproject.org) and RStudio version 1.3.1093 (https://www.rstudio.com).

To further identify sub-clusters within myeloid cell type, we re-analyzed cell type annotated as myeloid cells. Specifically, subset of Seurat object was extracted from the original expression matrix by default Seurat function, then we re-performed dimensionality reduction and PCA analysis and generate clusters as described above. Sub-cell types identified from sub-clustering of the myeloid cells were performed by “SingleR” package with “ImmGen” as the reference database, which identified Neutrophils, Macrophages, Monocytes, DC, Basophils cells. Finally, differentially expressed genes between PBS and *B.a* group in each sub-cell subsets were determined by Seurat package and visualized by “pheatmap” R packages.

### RNA sequencing

For RNA sequencing, BMDMs were co-cultured with *B.adolescentis* or vehicle (PBS) (MOI of 100:1) for 24 h. Total RNA was extracted from cells by Trizol reagent (Invitrogen, USA), and mRNA was purified by poly-T oligo-attached magnetic beads. Sequencing libraries were generated using NEBNext® Ultra™ RNA Library Prep Kit for Illumina® (NEB, USA) following manufacturer’s instruction. 150 bp paired-end libraries were sequenced by Illumina PE150 platform. Paired-end reads were aligned to the human genome version hg19 using Hisat2 v2.0.5. Differential expression analysis of two groups was performed using the DESeq2. *P* value < 0.05 and | log2 (fold change) |> 0.5 were considered as significant.

### Western blot

The Western blot was performed as previously described. The PVDF membranes were blocked with 5% skim milk for 1 h and then reacted with Decorin-specific antibody (Cat. #ab137508, diluted 1:1,000, Abcam), TLR2-specific antibody (Cat. #13744, diluted 1:1,000, CST), YAP-specific antibody (Cat. #13584-1-AP, diluted 1:1,000, Proteintech) at 4℃ overnight. Membranes were then incubated with Goat anti-Rabbit IgG-HRP (Cat. #HA1001, diluted 1:10,000, HUABIO) or Goat anti-Mouse IgG-HRP (Cat. #HA1006, diluted 1:10,000, HUABIO) at room temperature for 1 h. GAPDH (Cat. #60004-1-Ig, diluted 1:3,000, Proteintech) was used as a loading control.

### Histopathologic analysis

Colorectal tumors were fixed with 4% paraformaldehyde overnight at room temperature and embedded in paraffin. Hematoxylin and eosin (H&E) stain was performed as previously described.

For IHC, sections of paraffin-embedded tissue were stained by Ki67-specific antibody (Cat. #GB111141, diluted 1:1000, Servicebio), CD31-specific antibody (Cat. #GB11063-2, diluted 1:1000, Servicebio) and visualized by DAB staining (Cat. #G1212, Servicebio) according to the manufacturer’s instructions.

Immunofluorescence staining was performed as previously described. Tissue was incubated with DCN-specific antibody (Cat. #GB11300, diluted 1:1000, Servicebio) CD68-specific antibody (Cat. #GB14043, diluted 1:500, Servicebio) and F4/80-specific antibody (Cat. #GB14043, diluted 1:500, Servicebio). Respectively, sections were visualized by AF488-TSA (G1222, diluted 1:1000, Servicebio) and AF594-TSA (G1223, diluted 1:2000, Servicebio). Finally, sections were counterstained with 40,6-diamidino-2-phenylindole (DAPI; G1012, Servicebio) and imaged with NIKON digital sight DS-FI2 (NIKON Eclipse ci). For each section, the number of positive (F4/80^+^, CD68^+^, CD68^+^DCN^+^ and F4/80^+^DCN^+^) cells were analyzed in at least 5 randomly selected high-power views.

### Statistical analysis

Statistical analysis was performed using the GraphPad Prism 9.0 software. Data were analyzed with paired or unpaired Student *t* test, Mann–Whitney test, ANOVA test or linear regression as showed in figure legends. *P* value less than 0.05 was considered statistically significant.

## Results

### Supplementation of *B. adolescentis* suppressed colorectal tumorigenesis in mice

**Fig. 1:**
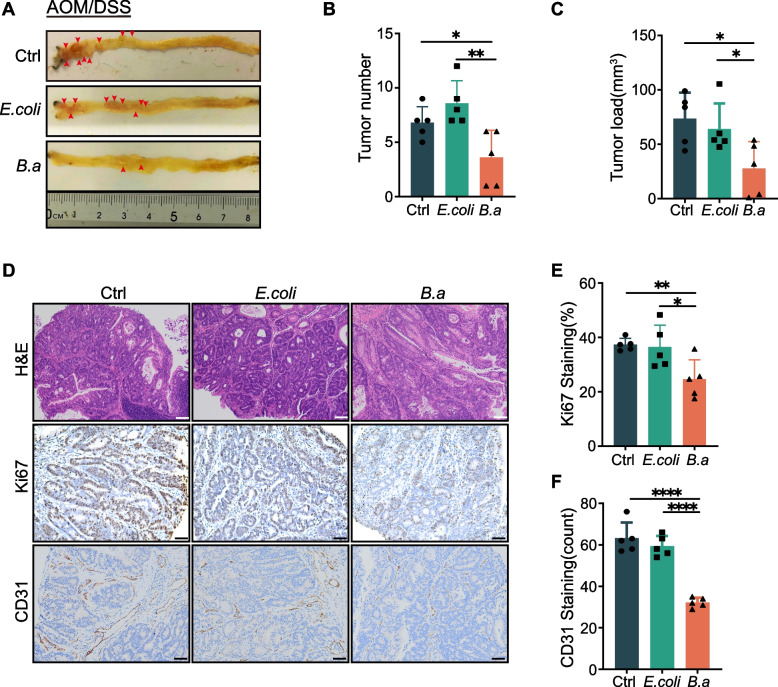
Supplementation of *B. adolescentis* suppressed colorectal tumorigenesis in mice. **A** Representative colorectum images of AOM/DSS mice treated with *B.adolescentis* (*B.a*; *n* = 5), *E. coli* (*n* = 5), or vehicle (PBS) (*n* = 5). The red arrows indicate the tumor locations. **B**, **C** Tumor number and load in the colorectum were measured. **D**-**F** Representative images and percentages of Ki67 and CD31 in colorectal cancer tissue by immunostaining; white scale bars, 200 μm; black scale bars, 50 μm. Data are shown as mean ± SD. * *P* < 0.05, ** *P* < 0.01, **** *P* < 0.0001; ANOVA test (**B**, **C**, **E**, **F**)

We observed that the abundance of *B.adolescentis* was significantly lower in tumor tissue from patients with CRC than in para-tumor (Supplementary Fig. 1A). To investigate the role of *B.adolescentis* in CRC, we utilized the AOM/DSS-induced CRC model in C57BL/6 mice, which were pretreated with streptomycin (2 mg/mL) for 7 days and administrated with *B.adolescentis*, *Escherichia coli* (*E. coli*) or vehicle (PBS) control (Supplementary Fig. 1B, C), and found that supplementation with *B.adolescentis* resulted in considerably fewer and smaller tumors than *E. coli* or vehicle group (Fig. 1A-C). The expression of cell proliferation marker Ki67 and tumor angiogenesis marker CD31 were lower in colorectal tumor tissue from *B.adolescentis*-treated mice (Fig. 1D-F). Collectively, these data indicated that *B.adolescentis* could suppress colorectal tumorigenesis in vivo.

### *B.adolescentis* recruited macrophages to suppress colorectal tumorigenesis

**Fig. 2:**
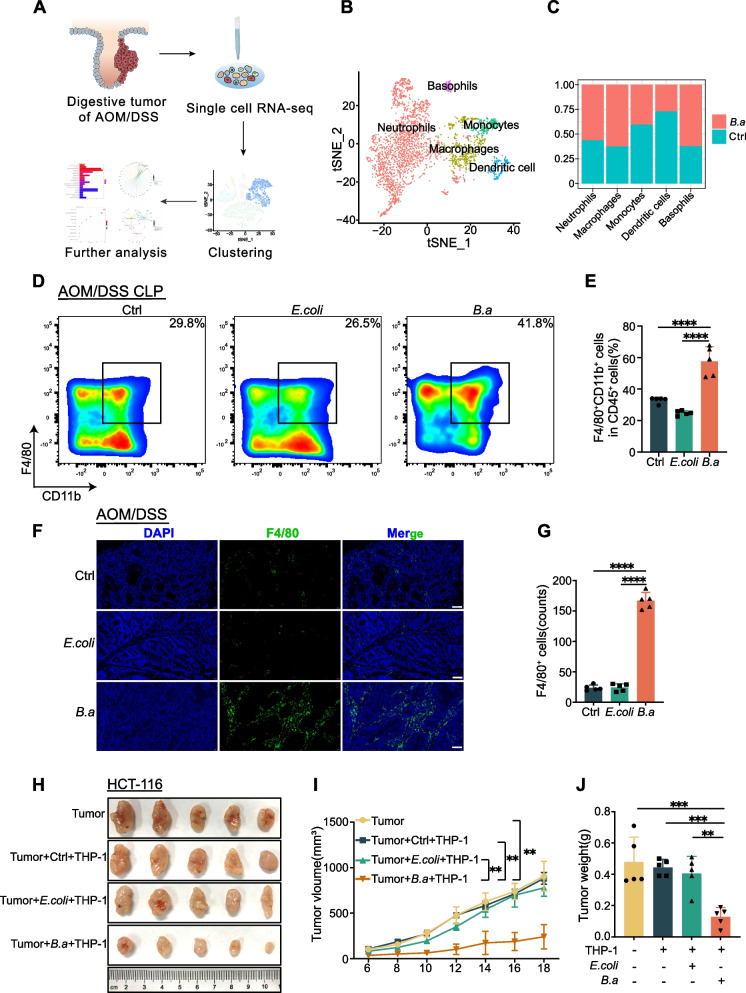
*B.adolescentis* recruited macrophages to suppress colorectal tumorigenesis. **A** Schematic representation of single cell preparation and scRNA-seq. **B** Myeloid cells were divided into 5 groups according to the top expression markers. **C** The percentage of 5 groups in *B.adolescentis* and vehicle (PBS) groups. **D**-**E** Flow cytometry representation and percentage of macrophages in colorectal lamina propria. **F**-**G** The number of F4/80^+^ cells was examined by immunofluorescence in the colorectal tumor of AOM/DSS mice treated with *B.adolescentis*, *E.coli* and vehicle (PBS); scale bars, 20 μm. **H**-**J** HCT116 cells were injected into BALB/c nude mice combined with THP-1 cells pretreated with *B.adolescentis*, *E. coli* or vehicle (PBS) for 24 h (*n* = 5 per group). Tumor volume and weight were recorded after 6 days. The independent experiment was repeated three times. Data are shown as mean ± SD. ** *P* < 0.01, *** *P* < 0.001, **** *P* < 0.0001; ANOVA test (**E**, **G**, **I**, **J**)

To understand the potential mechanism by which *B.adolescentis* suppresses CRC, we profiled the tumor from above AOM/DSS model using single-cell RNA sequencing (scRNA-seq) (Fig. 2A) and focused on the myeloid-derived immune cells (Supplementary Fig. 1D), as previous studies have shown that intratumoral *Bifidobacterium* facilitates innate immune response and scRNA-seq analyses informed the key role of myeloid-targeted therapies in CRC [16, 24]. We then divided myeloid-derived immune cells into five groups based on their top expression markers (Fig. 2B, Supplementary Fig. 1E) and found that *B.adolescentis* group exhibited an increase in the proportion of intratumoral macrophages and basophiles, and a decrease in dendritic cells (DCs) and monocytes (Fig. 2C, Supplementary Fig. 1F).

An increased percentage of macrophages was observed in colorectum lamina propria (CLP) and blood of AOM/DSS model treated with *B.adolescentis* when compared to *E.coli* or vehicle groups by flow cytometry assays (Fig. 2D, E, Supplementary Fig. 3A), whereas the percentage of DCs had no significant difference (Supplementary Fig. 3B). Similar results were confirmed in mice tumor tissue by immunofluorescence staining (Fig. 2F, G). Meanwhile, we co-cultured BMDMs or THP-1 cells with *B.adolescentis* for 24 h in vitro and found that *B.adolescentis-*treated macrophages exhibited a dramatic cell migration compared to *E.coli* or vehicle groups (Supplementary Fig. 3C, D).

To further determine the effect of *B.adolescentis*-treated macrophages on CRC progression, we injected HCT116 combined with *B.adolescentis*, *E. coli* or vehicle treated THP-1 cells (HCT116: THP-1 = 10:1) into BALB/c nude mice, and found that *B.adolescentis*-treated macrophages suppressed the tumor growth compared with *E. coli* or vehicle groups (Fig. 2H-J). In consideration of the extensive crosstalk among immune cells, the subcutaneous tumor experiment was performed in BALB/c immune-competent mice using CT26 and Raw264.7. *B.adolescentis*-treated macrophages consistently suppressed the tumor growth compared with *E. coli* or vehicle groups (Supplementary Fig. 3E-G). These data suggested that *B.adolescentis* promoted macrophages infiltration and had a suppressive effect on CRC.

### *B.adolescentis* facilitated the infiltration of Decorin^+^ macrophages to suppress CRC

**Fig. 3:**
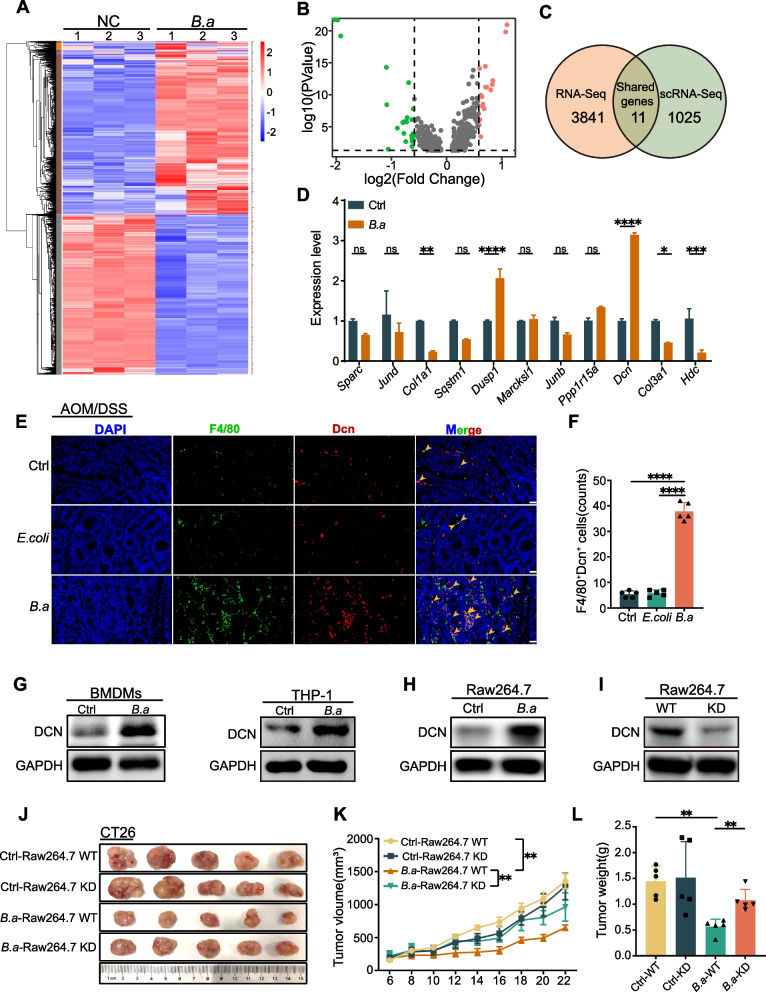
*B.adolescentis* facilitated the infiltration of Decorin^+^ macrophages to suppress CRC. **A** The heatmap of RNA-seq of macrophages treated with *B.adolescentis* or vehicle (PBS). **B** The volcano plot showed differentially expressed genes in scRNA-seq of macrophages treated with *B.adolescentis* or vehicle (PBS). **C** 3852 differentially expressed genes in RNA-seq and 1036 differentially expressed genes of macrophages in scRNA-seq were subjected to Venn diagram analysis (|log_2_fold change|> 0.5, p.adj < 0.05), and 11 shared differentially expressed genes were acquired. **D** The levels of 11 differentially expressed genes in BMDMs treated with *B.adolescentis* were determined by qRT-PCR. **E**, **F** The number of DCN^+^F4/80^+^ cells was examined by immunofluorescence in the colorectal tumor of AOM/DSS mice treated with *B.adolescentis, E. coli* or vehicle (PBS); The yellow arrows indicate the positively stained cells. scale bars, 20 μm. **G** BMDMs or human macrophage THP-1 cells were incubated with *B.adolescentis* or vehicle (PBS) for 24 h. Protein level of DCN was measured by Western blot. **H** Raw264.7 cells was incubated with *B.adolescentis* or vehicle (PBS) for 24 h. Protein level of DCN was measured by Western blot. **I **The protein level of DCN in Raw264.7 *Dcn*-knockdown cells or Raw264.7 control cells. **J**-**L** Raw264.7 *Dcn*-knockdown cells or control cells were pretreated with *B.adolescentis* or vehicle (PBS), and combined with CT26 were injected into BALB/c nude mice. Tumor volume was recorded after 6 days. The independent experiment was repeated three times. Data are shown as mean ± SD. ns: No statistical difference, * *P* < 0.05, ** *P* < 0.01, *** *P* < 0.001, **** *P* < 0.0001; Student *t* test (**D**), ANOVA test (**F**, **K**, **L**)

For exploration of the underlying mechanism by which *B.adolescentis*-treated macrophages exert anti-tumor function, we performed RNA-seq in BMDMs with or without *B.adolescentis* treatment (Fig. 3A), and its differentially expressed genes were analyzed and then overlapped with the differentially expressed genes of macrophages in vivo from the scRNA-seq mentioned above, and finally obtained 11 genes (Fig. 3B, C, Supplement Fig. 4A). We verified that *Decorin* (*Dcn*) had the highest fold change of expression in BMDMs treated with *B.adolescentis *in vitro (Fig. 3D). Furthermore, immunofluorescence staining showed that *B.adolescentis* increased the DCN^+^ macrophages (F4/80^+^DCN^+^) in tumor tissue of AOM/DSS model (Fig. 3E, F). Meanwhile, we analyzed the expression of *Dcn* in different cells subtypes in scRNA-seq, and found that macrophages exhibited higher *Dcn* expression compared to other cell subsets (Supplementary Fig. 4B). We found a significant increase in *Dcn*^+^ macrophages in *B.adolescentis* group through scRNA-seq data. (Supplementary Fig. 4C). Western blot analysis also confirmed that *B.adolescentis* increased the expression level of DCN in macrophages (Fig. 3G). Studies have reported that Dcn as a multivalent therapeutic agent against cancer by engaging multiple receptor tyrosine kinases like EGFR, Met and VEGFR2 [25], and Dcn deficiency promoted epithelial-mesenchymal transition and colorectum cancer metastasis [26]. To determine whether the tumor-suppressive effect of *B.adolescentis* on CRC relies on DCN^+^ macrophages, we established the *Dcn*-KD Raw264.7 cells by lentivirus-based Cas9 system (Fig. 3H, I). We then injected CT26 cells combined with *Dcn*-KD or wildtype Raw264.7 cells (CT26: Raw264.7 = 10:1) treated with or without *B.adolescentis* (MOI = 10:1) into BALB/c nude mice, and found that *B.adolescentis*-treated Raw264.7 obviously impeded tumor growth, whereas knockdown of *Dcn* notably diminished the tumor suppressive effect of *B.adolescentis* on CRC (Fig. 3J-L). We found *B.adolescentis*-treated macrophages had stronger cytotoxic activity against tumor cells than *E. coli* or vehicle (PBS) groups in vitro (Supplementary Fig. 4D, E). The levels of *Il6* and *Tnf* were elevated in *B.adolescentis*-treated macrophages, while *Dcn* deficiency did not influence the expression of *Il6* and *Tnf* (Supplementary Fig. 4F, G). Taken together, these data suggested that *B.adolescentis* induced DCN^+^ macrophages to suppress CRC growth.

### The activation of TLR2 is essential for inducing DCN^+^ macrophages by *B.adolescentis*

**Fig. 4:**
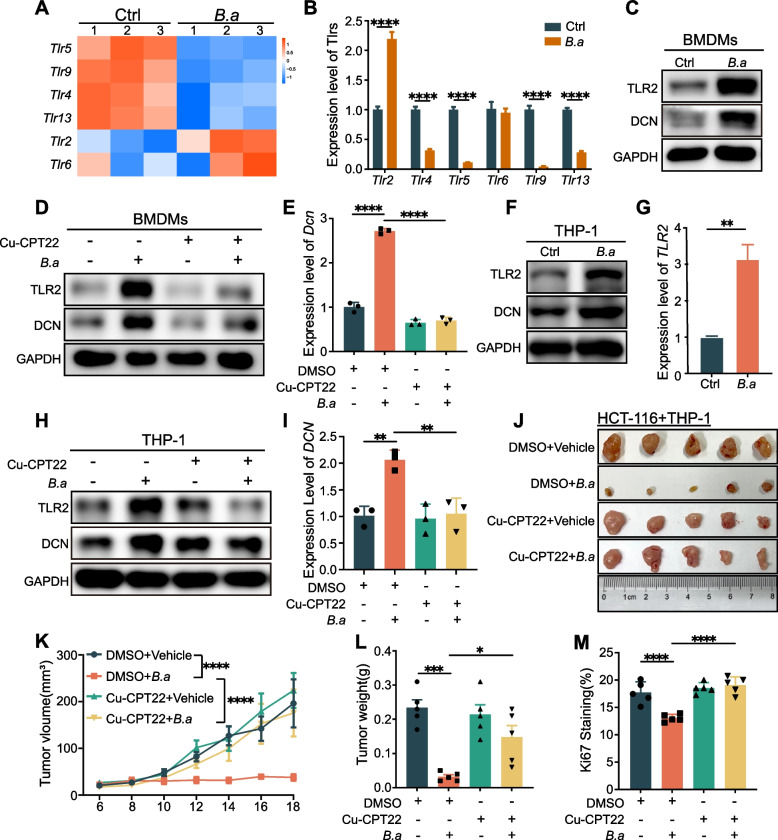
The activation of TLR2 is essential for inducing DCN^+^ macrophages by *B.adolescentis.* **A **The heatmap of differentially expressed TLRs genes in RNA-seq of BMDMs treated with *B.adolescentis* or vehicle (PBS). **B** The levels of differentially expressed TLRs genes in BMDMs treated with *B.adolescentis* were determined by qRT-PCR. **C** BMDMs were incubated with *B.adolescentis* or vehicle (PBS) for 24 h. Protein levels of TLR2 and DCN were tested by Western blot. **D**, **E** BMDMs were incubated with *B.adolescentis* or vehicle (PBS) for 24 h with or without 25 μM Cu-CPT22. Protein levels of TLR2 and DCN were tested by Western blot and mRNA level of *Dcn* was tested by qRT-PCR. **F**-**I** THP-1 cells were incubated with *B.adolescentis* or vehicle (PBS) for 24 h (**H**-**G**). THP-1 cells were incubated with *B.adolescentis* or vehicle (PBS) for 24 h with or without 25 μM Cu-CPT22 (**H**, **I**). Protein levels of TLR2 and DCN were tested by Western blot and mRNA level of *DCN* was tested by qRT-PCR. **J**-**L** HCT116 cells were injected into BALB/c nude mice combined with THP-1 cells pretreated with *B.adolescentis* or vehicle (PBS) for 24 h (*n* = 5 per group). From the beginning of tumor inoculation until sacrifice, 3 mg/kg Cu-CPT22 or vehicle (5% DMSO) were injected intraperitoneally to mice every two days. Tumor volume was recorded after 6 days. **M** The positive ratio of Ki67 in mice tumor tissue. The independent experiment was repeated three times. Data are shown as mean ± SD. * *P* < 0.05, ** *P* < 0.01, *** *P* < 0.001, **** *P* < 0.0001; Student *t* test (**B**, **G**), ANOVA test (**E**, **I**, **K**, **L**, **M**)

Pattern recognition receptors (PRRs) play a central role in innate immune response [27, 28]. As Toll-like receptors (TLRs) are a major class of PPRs that recognize extracellular signals, we screened out TLRs that were differentially expressed in RNA-seq of *B.adolescentis*-treated BMDMs (Fig. 4A) and checked them by qRT-PCR, and discovered that *TLR2* was most significantly upregulated (Fig. 4B). We then confirmed the upregulation of TLR2 in BMDMs treated with *B.adolescentis* at the protein level (Fig. 4C). Mechanically, to verify that *B.adolescentis* induced the DCN^+^ macrophages via TLR2, we performed TLR2 inhibition assays by Cu-CPT22 and found that Cu-CPT22 administration abolished the upregulation of DCN in BMDMs treated with *B.adolescentis* (Fig. 4D, E). Similar results were reproduced in THP-1 cells (Fig. 4F-I). We further combined injected HCT116 and THP-1 cells with *B.adolescentis *(MOI = 10:1) or vehicle into BALB/c nude mice along with intraperitoneally injecting Cu-CPT22 every two days. We found that TLR2 inhibition reduced the number of DCN^+^ macrophages in tumor and attenuated tumor suppressive effect of *B.adolescentis*-treated macrophages (Fig. 4J-M, Supplementary Fig. 5A-C). These results suggested that *B.adolescentis* induced the DCN^+^ macrophages in a TLR2-dependent way.

### *B.adolescentis* regulated DCN^+^ macrophages through TLR2/YAP axis

**Fig. 5:**
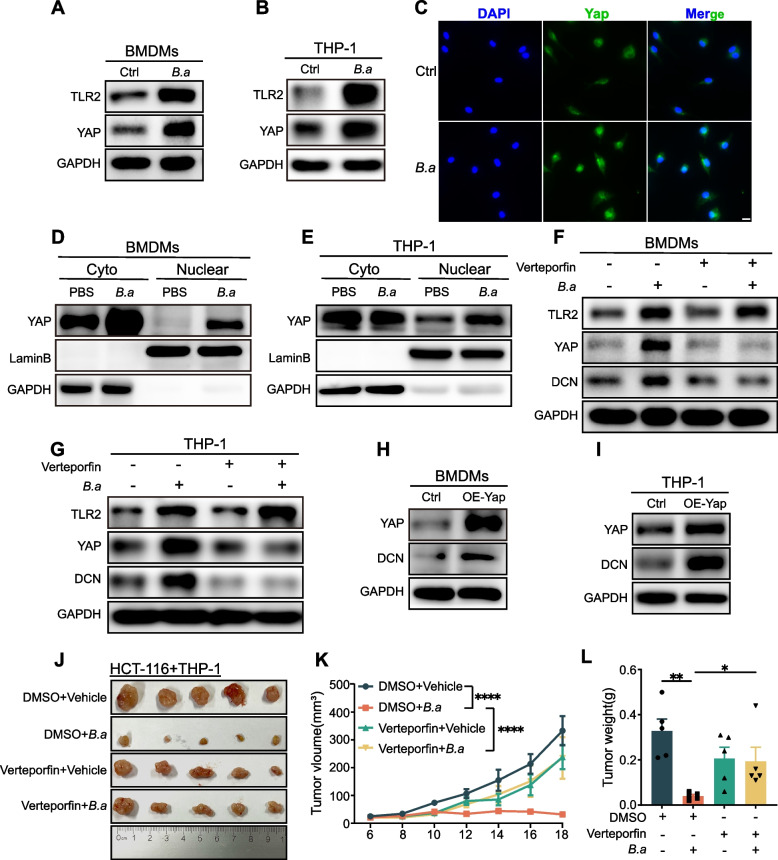
*B.adolescentis* regulated DCN^+^ macrophages through TLR2/YAP axis. **A**, **B** BMDMs and THP-1 cells were incubated with *B.adolescentis* or vehicle (PBS) for 24 h. Protein levels of TLR2 and YAP were tested by Western blot. **C** Immunofluorescence assay of YAP distribution in BMDMs. BMDMs were stained with specific antibody against YAP (green), and the nuclei were counterstained with DAPI (blue). Scale bar, 10 μm. **D**, **E** Nuclear and cytoplasmic separation assay was performed in *B.adolescentis-*treated BMDMs and THP-1 cells, then the protein level of YAP in the nuclear and cytoplasm was detected by Western blot. **F**, **G** BMDMs and THP-1 cells were incubated with *B.adolescentis* or vehicle (PBS) for 24 h with or without 1 μM verteporfin. Protein levels of TLR2, YAP and DCN were tested by Western blot. **H**, **I** YAP was overexpressed in BMDMs and THP-1 cells, and protein levels of YAP and DCN were tested by Western blot. **J**-**L** HCT116 cells were injected into BALB/c nude mice combined with THP-1 cells pretreated with *B.adolescentis* or vehicle (PBS) for 24 h (*n* = 5 per group). From the beginning of tumor inoculation until sacrifice, 50 mg/kg verteporfin or vehicle (5% DMSO) were injected intraperitoneally to mice every day. Tumor volume was recorded after 6 days. The independent experiment was repeated three times. Data are shown as mean ± SD. * *P* < 0.05, ** *P* < 0.01, **** *P* < 0.0001; ANOVA test (**K**, **L**)

Previous studies have demonstrated that TLR2/YAP axis regulated cancer innate immunity [29, 30], yet the function of TLR2/YAP axis in macrophages was unclear. We discovered that the level of YAP was upregulated in BMDMs and THP-1 cells (Fig. 5A, B) and TLR2 inhibition diminished the regulation of *B.adolescentis* on YAP (Supplementary Fig. 6A). The nuclear expression of YAP increased in BMDMs and THP-1 cells after *B.adolescentis* treatment (Fig. 5C-E). YAP inhibitor verteporfin markedly reduced the expression of YAP and DCN in *B.adolescentis*-treated macrophages but it did not influence the expression of TLR2 (Fig. 5F, G, Supplementary Fig. 6B, C). And overexpression of YAP in BMDMs and THP-1 cells increased the level of DCN (Fig. 5H, I). We then injected HCT116 combined with *B.adolescentis* (MOI = 10:1) or vehicle treated THP-1 cells into BALB/c nude mice, and intraperitoneally injected with verteporfin every days. YAP inhibitor administration attenuated the tumor suppressive effect of *B.adolescentis*-treated THP-1 cells and reduced the number of DCN^+^ macrophages in tumors on BALB/c nude mice. (Fig. 5J-L, Supplementary Fig. 6D-F). Furthermore, we found that the expression levels of DCN, TLR2 and YAP were elevated after treated with *B.adolescentis* in human primary macrophages (Supplementary Fig. 7A, B).

To investigate the relationship between DCN^+^ macrophages and macrophage polarization state, we examined the related markers of macrophages after co-culturing with *B.adolescentis* and found that the number of M1 macrophages increased while M2 macrophages decreased in *B.adolescentis*-treated BMDMs (Supplementary Fig. 8A-D). *B.adolescentis* upregulated the expression levels of DCN, TLR2 and YAP in M1 macrophages (Supplementary Fig. 8E, F), which reflected that the function of DCN^+^ macrophages might be similar with traditional M1-like macrophages*.* These results reflected that *B.adolescentis* increased DCN^+^ macrophages through the TLR2/YAP axis.

### *B.adolescentis* abundance correlated with TLR2 and DCN^+^ macrophages in patients with CRC

**Fig. 6:**
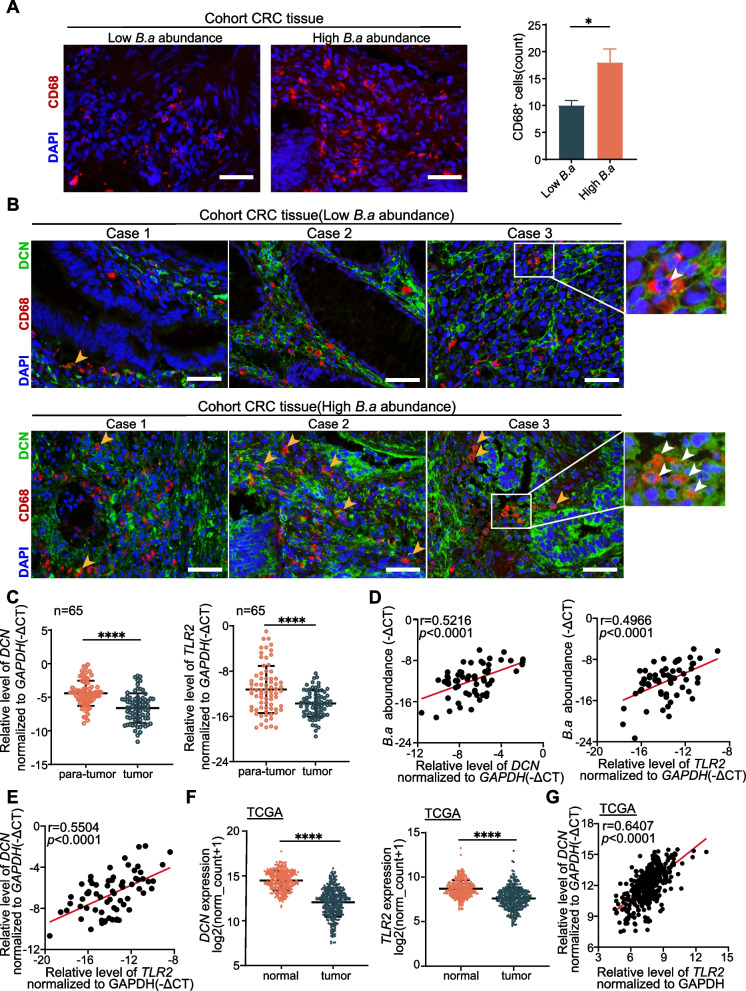
*B.adolescentis* abundance correlated with TLR2 and DCN^+^ macrophages in patients with CRC. **A** Representative images of immunofluorescence assay for CD68 in tumor tissue with low or high *B.adolescentis* abundance from cohort; scale bars, 50 μm. **B** Representative images of immunofluorescence staining of DCN^+^ macrophages (CD68^+^DCN^+^ dual positive) in tumor tissue of CRC; scale bars, 50 μm. The yellow and white arrows indicate the positively stained cells. **C** qRT-PCR analysis of *DCN* and *TLR2* mRNA expression in tumor or para-tumor tissue of CRC. **D** The correlation of *B.adolescentis* with *DCN* or *TLR2* was analyzed in tumor tissue of CRC. **E** The correlation of *DCN* and *TLR2* was analyzed in tumor tissue of CRC. **F** Transcript levels of *DCN* and *TLR2* in colorectal cancer and normal tissue from TCGA database. **G** The correlation of *DCN* and *TLR2* was analyzed in TCGA database. The independent experiment was repeated three times. Data are shown as mean ± SD. * *P* < 0.05, **** *P* < 0.0001; Mann–Whitney test (**A**, **C**, **F**), Linear Regression (**D**, **E**, **G**)

To further investigate the clinical relevance, we quantified *B.adolescentis* and the expression of *TLR2* and *DCN* with quantitative qRT-PCR analysis in paired fresh CRC and adjacent non-tumor tissue. According to the level of *B.adolescentis* in CRC tissue, we divided these patients (*n* = 65) into high and low *B.adolescentis* group. We found that compared with the low *B.adolescentis* group, the number of macrophages in high *B.adolescentis* group was higher (Fig. 6A), and the abundance of *B.adolescentis* was related to the number of DCN^+^ macrophages (Fig. 6B). Moreover, the expression of *DCN* and *TLR2* was decreased in CRC tissue compared with their adjacent non-tumor tissue (Fig. 6C), and the abundance of *B.adolescentis* was positively correlated with the expression of *TLR2* and *DCN* in CRC tissue (Fig. 6D). In addition, the expression of *DCN* was correlated with the expression of *TLR2* (Fig. 6E). Lastly, we confirmed the above results using TCGA GTEx datasets (Fig. 6F, G). These data indicated that *B.adolescentis* abundance correlated with the expression of *TLR2* and the number of DCN^+^ macrophages in patients with CRC.

## Discussion

Several infant-type species of human-derived bifidobacterial (HRB) are associated with strengthening host immunity and suppressing tumorigenesis. However, the role and mechanism of adult-type HRB in cancer remains unclear [31]. In this study, we revealed that *B.adolescentis* suppressed colorectal tumorigenesis in AOM/DSS mice.

Macrophages are a major component of TME whose functional plasticity leads to anti-tumor and pro-tumor function in different environment [32]. Furthermore, targeting macrophages is an emerging field of interest due to synergies with immune checkpoint inhibitors, chemotherapy, and radiation therapy in preclinical studies [33, 34]. Our groups have reported that some specific microbes were closely related to macrophages functions [20, 35]. In this study, we discovered that *B.adolescentis* suppressed colorectal tumorigenesis through inducing DCN^+^ macrophages, and targeting macrophages with *B.adolescentis* maybe a potential novel strategy for cancer therapy.

Decorin is a member of the small leucine-rich proteoglycans (SLRPs) which are an important subset of extracellular matrix [36]. Soluble DCN engages multiple receptor tyrosine kinases within the target-rich environment of the tumor stroma and tumor parenchyma [25]. DCN possesses a multitude of oncosuppressive functions including growth suppressing, angiostasis, and tumor cells mitophagy arrest [37]. Several research groups have discovered that DCN deficiency promotes epithelial-mesenchymal transition and hepatic metastasis of colorectal carcinoma [26, 37]. DCN derived from T cells mediates inhibition of carcinogenesis in microglia and reduces glioma formation [38]. However, the function of macrophage-derived DCN has not been reported. We found that *B.adolescentis* increased the DCN^+^ macrophages and then suppressed colorectal tumorigenesis. *B.adolescentis* also boosted the cytotoxicity of macrophages against tumor cells and increased the expression of *Il6* and *Tnf* in macrophages in a *Dcn*-independent way.

Toll-like receptors (TLRs) are an important component family of pattern recognition receptors (PRRs) responsible for early recognition in the innate immune system. TLR2 recognizes a vast of ligands derived from microbes such as lipoproteins/lipopeptides and peptidoglycans [39]. *Bifidobacterium breve* has been reported to induce dendritic cell maturation, activation, and survival via TLR2 [40]. Here, we found that *B.adolescentis* elevated the expression of TLR2 in macrophages and the activation of TLR2 is essential for *B.adolescentis* regulating DCN^+^ macrophages. However, how *B.adolescentis* was recognized by TLR2 in macrophages needs to be further explored.

Emerging evidences suggested that the differentiation, metabolism, and functions of innate immune cells were extensively regulated by YAP [41, 42]. Microbes are reported to regulate the activation of YAP in cancer cells to inhibits colorectal tumorigenesis [43, 44]. YAP in macrophages promoted the LPS/IFN-γ-triggered activation of M1 macrophages, while impaired the IL-4/IL-13 induced M2 macrophages polarization [45]. Prior studies suggested that TLR2/YAP axis played an important role in innate immunity and the activation of TLR2/YAP axis in tumor cells suppressed tumor growth [29, 30]. However, the underlying mechanism of *B.adolescentis* interacted with macrophages and the function of TLR2/YAP axis in macrophages remain largely unknown. Indeed, we observed that TLR2/YAP axis was activated by *B.adolescentis* and inhibition of TLR2/YAP axis significantly impaired the induction of DCN^+^ macrophages by *B.adolescentis*. Consistent with our findings, *B.adolescentis, TLR2* and *DCN* were reduced in CRC tissue. However, the relationship between the expression of *TLR2* and *DCN* in macrophages and the abundance of *B.adolescentis* needs to be further explored.

In conclusion, our results provided a novel insight into the interaction and molecular mechanism of *Bifidobacterium* with macrophages. Our findings suggested that *B.adolescentis* could act as probiotics to regulate macrophages in colorectal cancer, thus becoming a potential target for tumor vaccine delivery and providing a novel strategy for tumor therapy.

### Supplementary Information

**Additional file 1: Figure S1. **Supplementation of *B. adolescentis *suppressed colorectal tumorigenesis and increased infiltration of macrophages. **Figure S2. **Gating strategy of macrophages and dendritic cells. **Figure S3.*** B.adolescentis* recruited macrophages to suppress colorectal tumorigenesis. **Figure S4.*** B.adolescentis *facilitated the infiltration of Decorin^+^ macrophages to suppress CRC. **Figure S5.**The activation of TLR2 is essential for inducing DCN^+^ macrophages by *B.adolescentis*. **Figure S6. ***B.adolescentis* regulated DCN^+^ macrophages through TLR2/YAP axis. **Figure S7.*** B.adolescentis *activated TLR2/YAP/DCN in primary human macrophages. **FigureS8. ***B.adolescentis* activated TLR2/YAP/DCN in M1 macrophages. **Table S1. **Primers used for validation the gene expression level. 

## Data Availability

All data generated or analyzed during the study are included in this published article and its additional files.

## Abbreviations

*B.a*: *Bifidobacterium adolescentis*
CRC: Colorectal cancer
qRT-PCR: Quantitative real-time PCR
TME: Tumor microenvironment
DCN: Decorin
