# Correction: *Bifidobacterium adolescentis* induces Decorin^+^ macrophages via TLR2 to suppress colorectal carcinogenesis

**DOI:** 10.1186/s13046-023-02796-w

**Published:** 2023-08-19

**Authors:** Yifeng Lin, Lina Fan, Yadong Qi, Chaochao Xu, Dingjiacheng Jia, Yao Jiang, Shujie Chen, Liangjing Wang

**Affiliations:** 1 Department of Gastroenterology, Second Affiliated Hospital of Zhejiang University School of Medicine, Hangzhou, 310009 Zhejiang Province China; 2 Institute of Gastroenterology, Zhejiang University, Hangzhou, China; 3 Department of Gastroenterology, School of Medicine, Sir Run Run Shaw Hospital, Zhejiang University, Hangzhou, 310003 Zhejiang Province China; 4 Cancer Center, Zhejiang University, Hangzhou, Zhejiang China; 5 Research Center of Prevention and Treatment of Senescent Disease, School of Medicine, Zhejiang University, Hangzhou, China

**Correction:** ***J Exp Clin Cancer Res*** **42, 172 (2023)**

**https://doi.org/10.1186/s13046-023-02746-6**

Following publication of the original article [1], authors identified an error in Fig. 4F. The images in Fig. 4F are mistakenly pasted.

The corrected Fig. 4 is presented below:

**Fig. 4:**
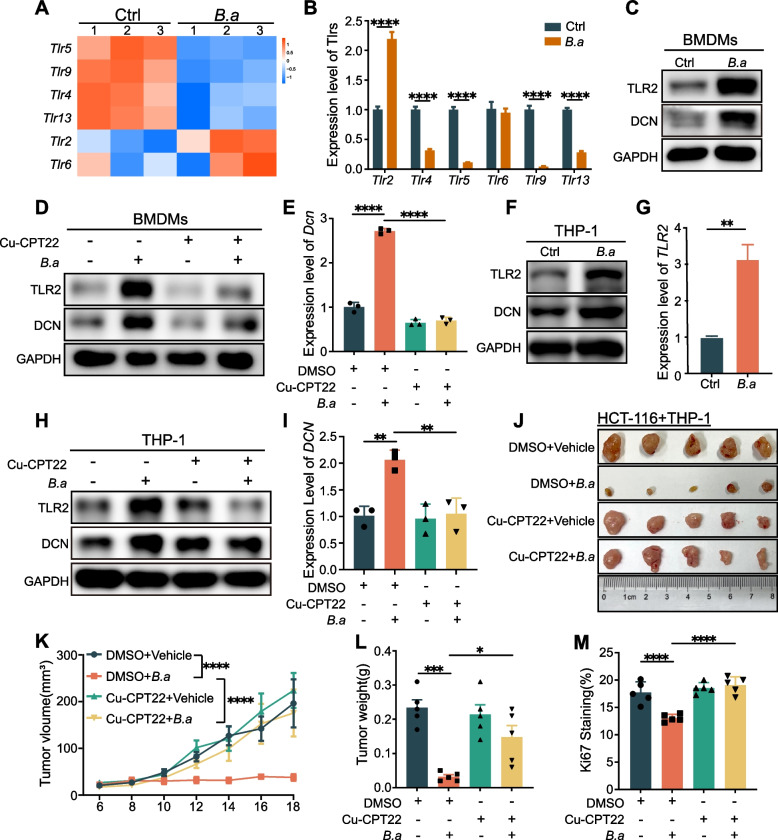
The activation of TLR2 is essential for inducing DCN^+^ macrophages by *B.adolescentis.* **A** The heatmap of differentially expressed TLRs genes in RNA-seq of BMDMs treated with *B.adolescentis* or vehicle (PBS). **B** The levels of differentially expressed TLRs genes in BMDMs treated with *B.adolescentis* were determined by qRT-PCR. **C** BMDMs were incubated with *B.adolescentis* or vehicle (PBS) for 24 h. Protein levels of TLR2 and DCN were tested by Western blot. **D-E** BMDMs were incubated with *B.adolescentis* or vehicle (PBS) for 24 h with or without 25uM Cu-CPT22. Protein levels of TLR2 and DCN were tested by Western blot and mRNA level of *Dcn* was tested by qRT-PCR. (**F-I**) THP-1 cells were incubated with *B.adolescentis* or vehicle (PBS) for 24 h (**H-G**). THP-1 cells were incubated with *B.adolescentis* or vehicle (PBS) for 24 h with or without 25uM Cu-CPT22 (**H-I**). Protein levels of TLR2 and DCN were tested by Western blot and mRNA level of *DCN* was tested by qRT-PCR. **J-L** HCT116 cells were injected into BALB/C nude mice combined with THP-1 cells pretreated with *B.adolescentis* or vehicle (PBS) for 24 h (*n* = 5 per group). From the beginning of tumor inoculation until sacrifice, 3 mg/kg Cu-CPT22 or vehicle (5% DMSO) were injected intraperitoneally to mice every two days. Tumor volume was recorded after 6 days. **M** The positive ratio of Ki67 in mice tumor tissue. The independent experiment was repeated three times. Data are shown as mean ± SD. * *P* < 0.05, ** *P* < 0.01, *** *P* < 0.001, **** *P* < 0.0001; Student *t* test (**B, G**), ANOVA test (**E, I, K, L, M**)

The correction does not affect the overall conclusion of the article. The original article has been corrected.
