# Supplementary material for: Bifidobacterium adolescentis induces Decorin+ macrophages via TLR2 to suppress colorectal carcinogenesis
Source: J Exp Clin Cancer Res. 2023 Jul 18;42:172. doi: 10.1186/s13046-023-02746-6 (PMC10353206; doi:10.1186/s13046-023-02746-6)
Supplement: Supplementary file 1 — Additional file 1: Figure S1. Supplementation of B. adolescentis suppressed colorectal tumorigenesis and increased infiltration of macrophages. Figure S2. Gating strategy of macrophages and dendritic cells. Figure S3. B.adolescentis recruited macrophages to suppress colorectal tumorigenesis. Figure S4. B.adolescentis facilitated the infiltration of Decorin+ macrophages to suppress CRC. Figure S5.The activation of TLR2 is essential for inducing DCN+ macrophages by B.adolescentis. Figure S6. B.adolescentis regulated DCN+ macrophages through TLR2/YAP axis. Figure S7. B.adolescentis activated TLR2/YAP/DCN in primary human macrophages. FigureS8. B.adolescentis activated TLR2/YAP/DCN in M1 macrophages. Table S1. Primers used for validation the gene expression level. [file 13046_2023_2746_MOESM1_ESM.docx]

**Supplementary Materials**


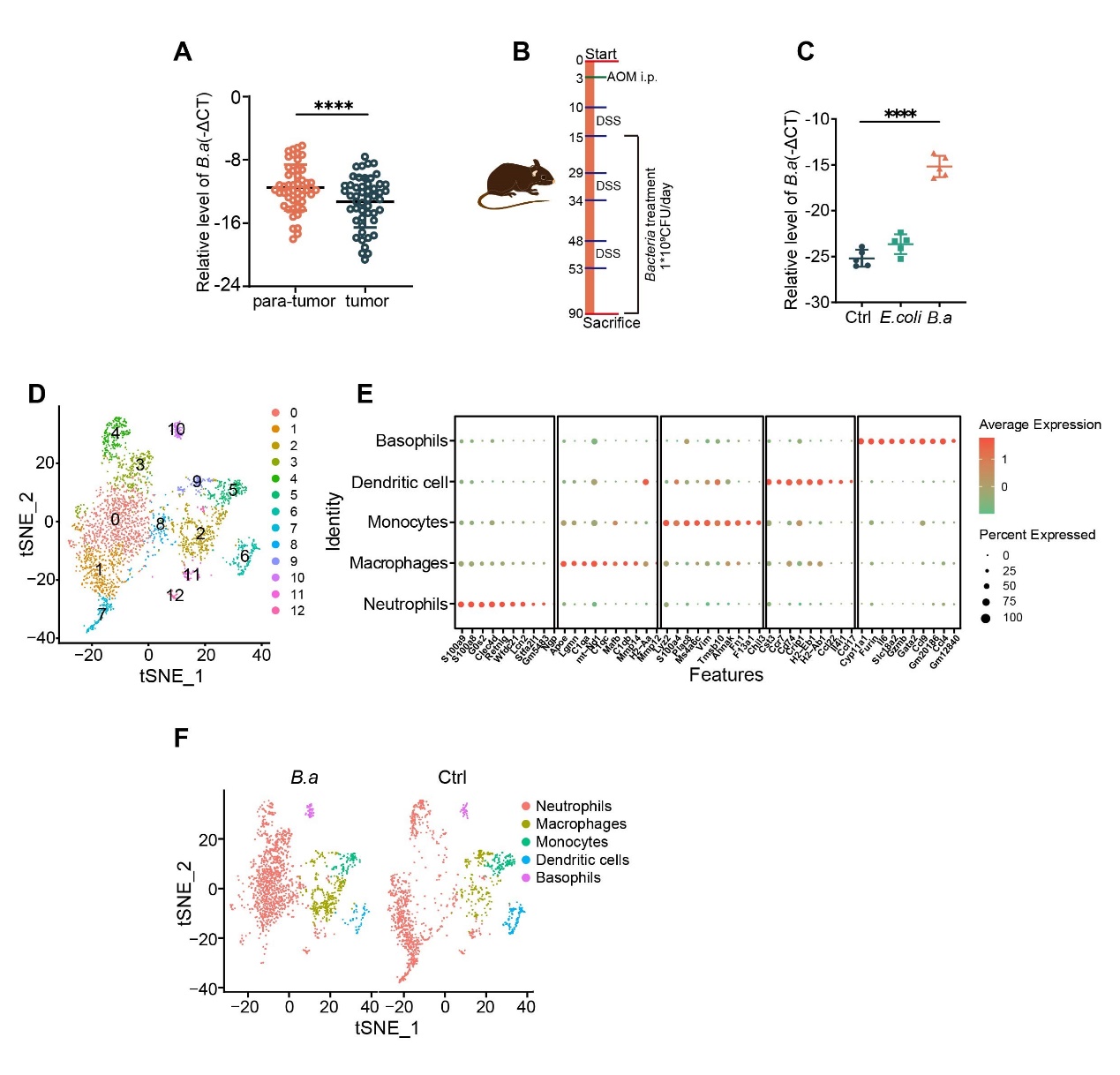


**Figure S1. Supplementation of *B. adolescentis* suppressed colorectal tumorigenesis and increased infiltration of macrophages.** (A) The relative abundance of *B.adolescentis* was determined in tumor or para-tumor tissue of CRC by qRT-PCR. (B) Experimental model of AOM/DSS mice model. (C) The relative abundance of *B.adolescentis* was determined by qRT-PCR in feces of AOM/DSS mice. (D) tSNE plots of scRNA-seq data from tumor-infiltrating myeloid cells after treatment with *B.adolescentis* or vehicle (PBS). (E) The top expression markers of myeloid cells. (F) tSNE plot of scRNA-seq data from tumor-infiltrating myeloid cells of *B.adolescentis* or vehicle (PBS) groups. The independent experiment was repeated three times. Data are shown as mean ± SD. **** P < 0.0001, Wilcoxon matched-pairs signed-rank test (A), ANOVA test(C).


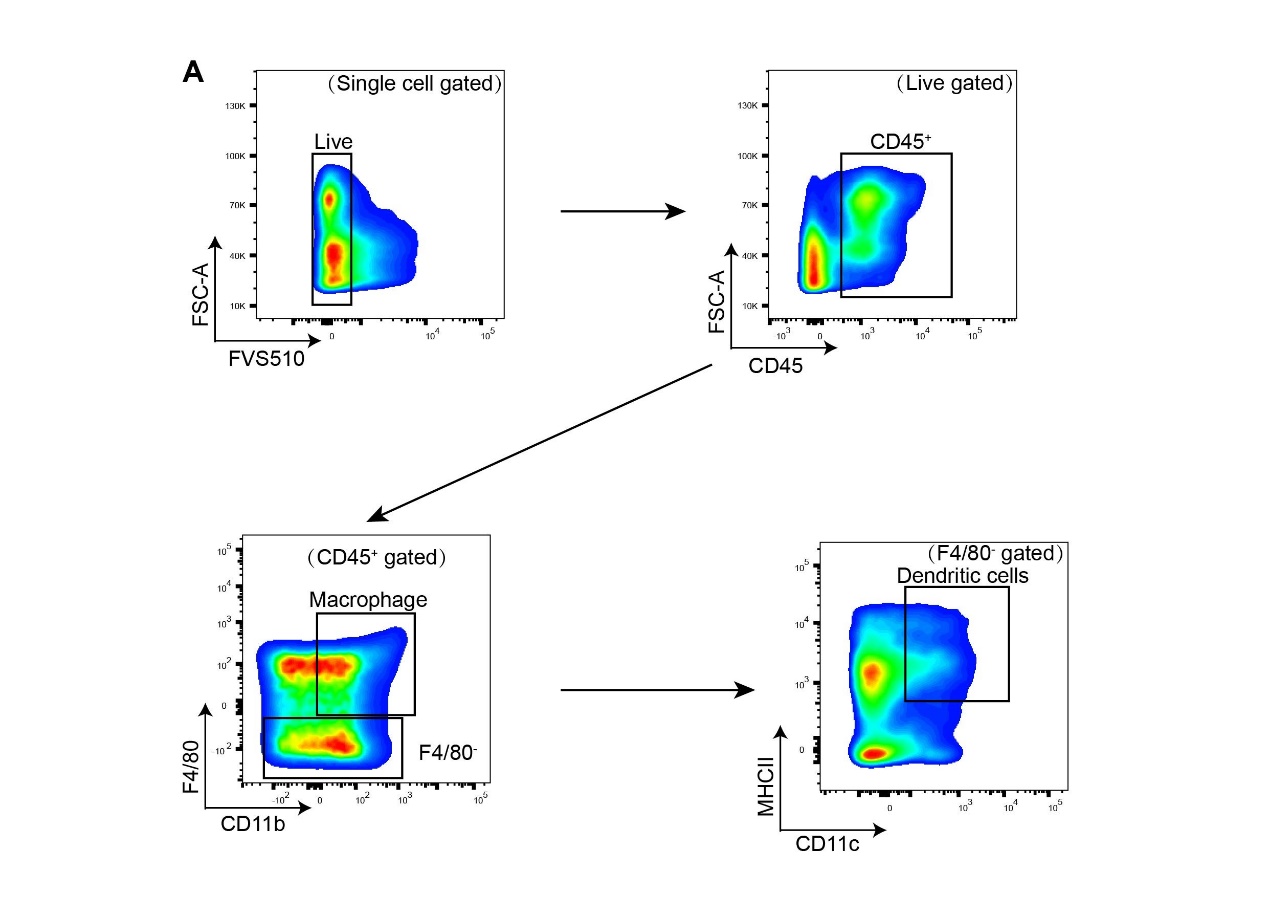


**Figure S2. Gating strategy of macrophages and dendritic cells.** (A) We utilized Fixable viability stain 510 to gate the live cells (FVS^-^ cells) gated on single cells. Then we identified the immune cells by CD45 gated on live cells. Macrophages were determined by F4/80 and CD11b gated on CD45^+^ cells and dendritic cells were determined by CD11c and MHCII gated on CD45^+^F4/80^-^ cells.


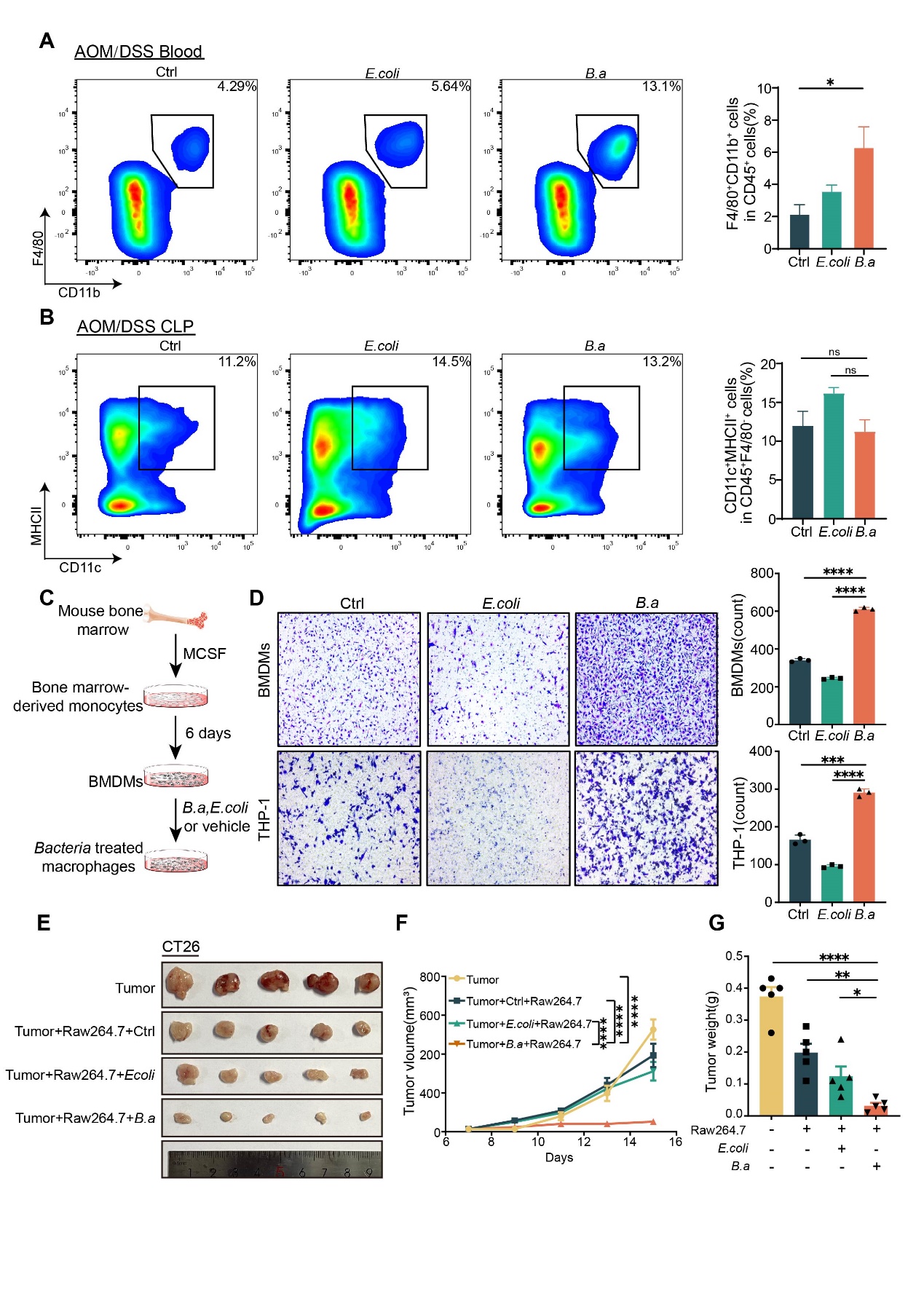


**Figure S3. *B.adolescentis* recruited macrophages to suppress colorectal tumorigenesis.** (A) Flow cytometry representation and percentage of macrophages in blood of AOM/DSS mice model. (B) Flow cytometry representation and percentage of dendritic cells in colorectal lamina propria of AOM/DSS mice model. (C) Schematic representation of BMDMs isolation and co-cultured with *B.adolescentis*, *E. coli* or vehicle (PBS). (D) The migration assay of BMDMs and THP-1 cells pretreated with *B.adolescentis*, *E.coli* or vehicle (PBS) for 24 hours. The migrated cells were quantified at 24 hours by counting in five fields. Scale bar, 100 μm. (E-G) Mouse macrophages Raw264.7 cells were pretreated with *B.adolescentis*, *E.coli* or vehicle (PBS), and injected into BALB/c mice combined with CT26 cells. Tumor volume and weight were recorded after 7 days. The independent experiment was repeated three times. Data are shown as mean ± SD. * *P* < 0.05, ** P < 0.01, **** P < 0.0001; ANOVA test (A, B, D, F, G).


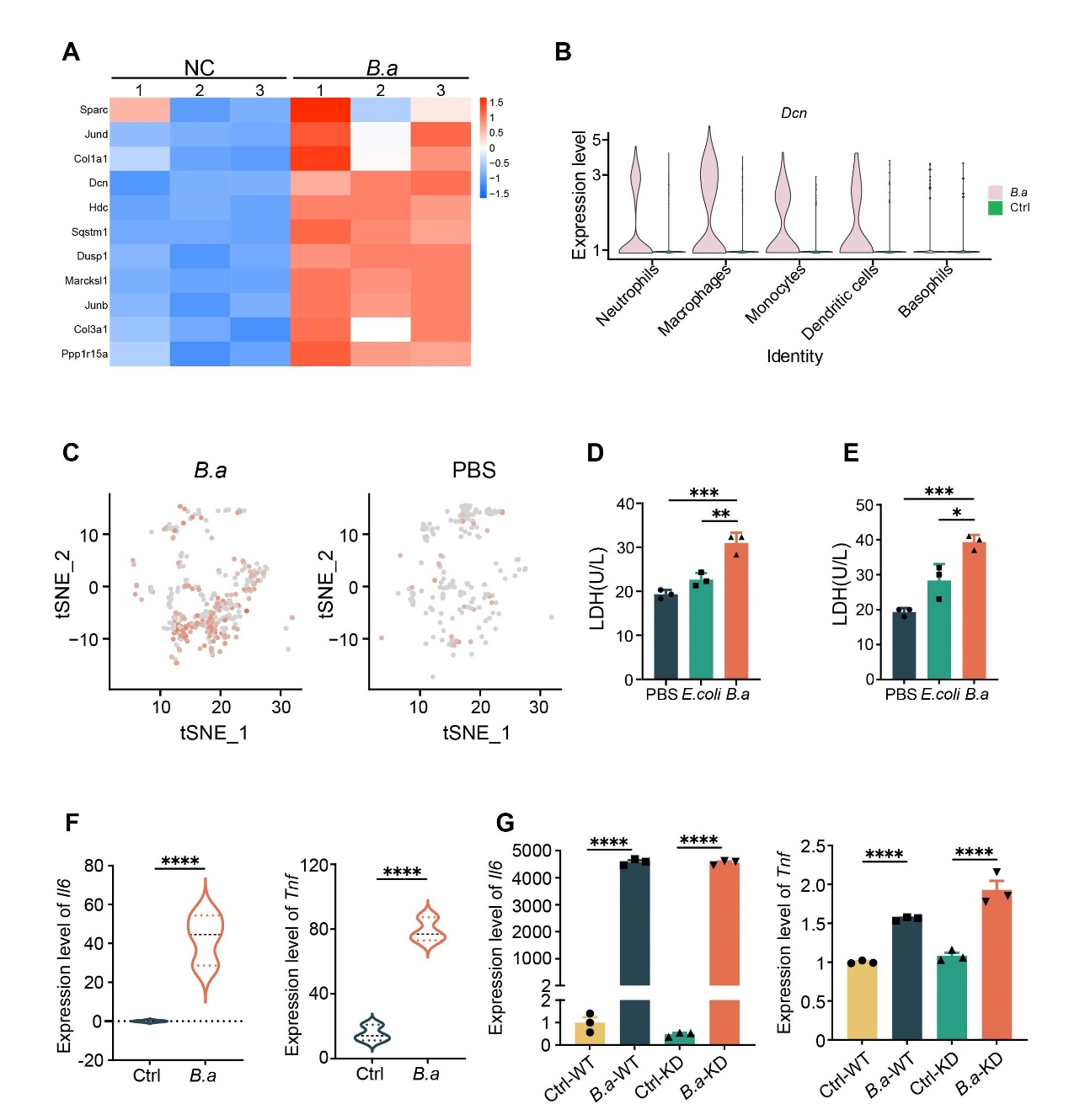


**Figure S4. *B.adolescentis* facilitated the infiltration of Decorin^+^ macrophages to suppress CRC*.*** (A) The heatmap of the 11 differentially expressed genes overlapped by differentially expressed genes of RNA-seq and macrophages in scRNA-seq. (B) The violin plots of the expression level of *Dcn* in scRNA-seq of different myeloid groups cells. (C) tSNE plots in scRNA-seq data of tumor-infiltrating DCN^+^ macrophages of *B.adolescentis* or vehicle (PBS) groups in AOM/DSS model. The orange dots represent DCN^+^ macrophages. (D-E) BMDMs pretreated with *B.adolescentis*, *E. coli* or vehicle (PBS) *in vitro* for 24 hours. And then the LDH in the medium was tested after BMDMs co-cultured with HCT116 (D) or CT26 (E) (BMDMs: cancer cells = 1:1) for 24 hours. (F) The levels of *Il6* and *Tnf* in RNA-seq of BMDMs treated with *B.adolescentis* or vehicle (PBS)*.* (G) The levels of *Il6* and *Tnf* in WT or *Dcn*-KD Raw264.7 treated with *B.adolescentis* were determined by qRT-PCR. The independent experiment was repeated three times. Data are shown as mean ± SD, * *P* < 0.05, ** P < 0.01, *** P < 0.001, **** P < 0.0001; ANOVA test (D, E, G), Student *t* test (F).


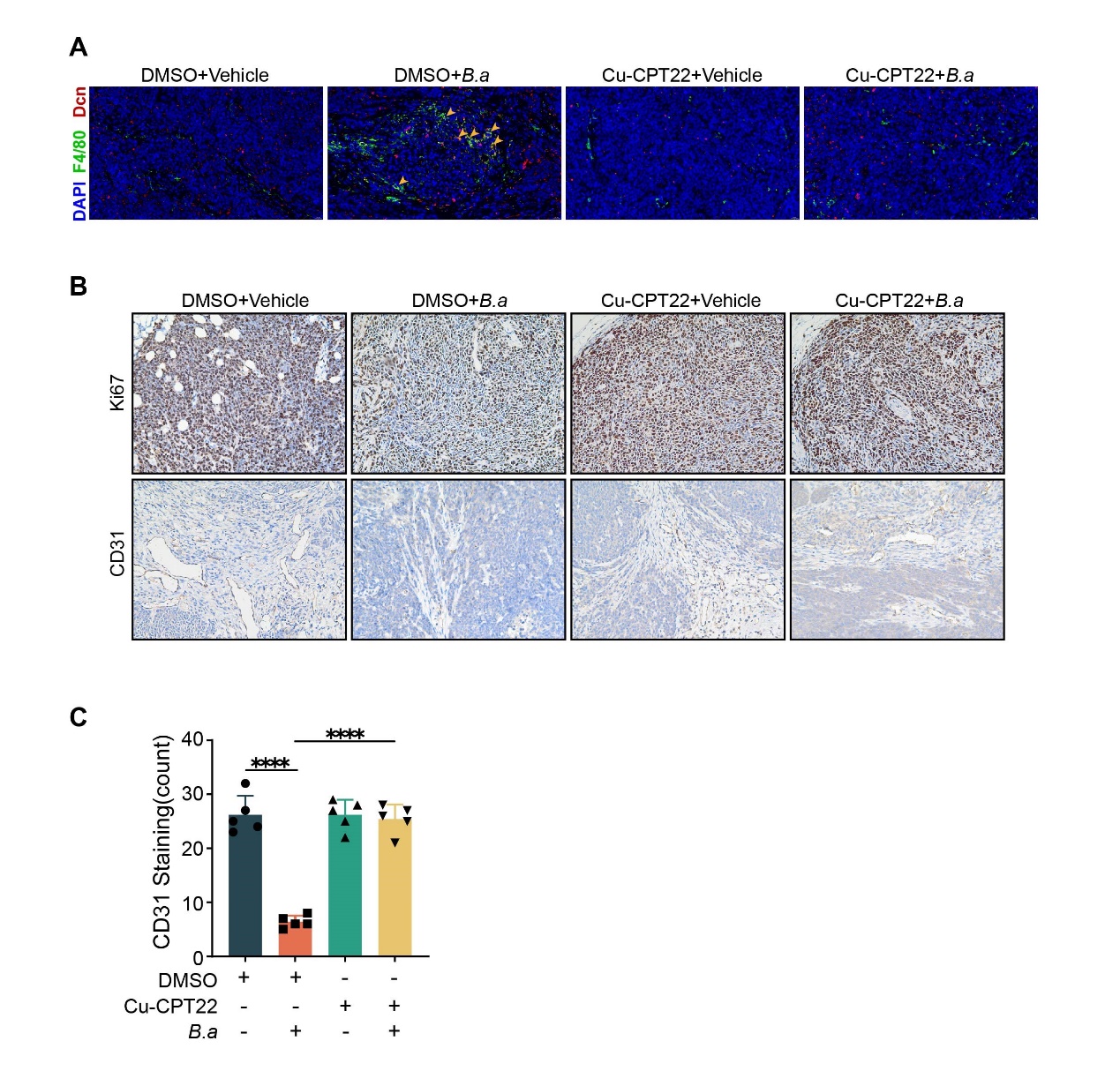


**Figure S5.** **The activation of TLR2 is essential for inducing DCN^+^ macrophages by *B.adolescentis.*** (A) The number of DCN^+^F4/80^+^ cells was examined by immunofluorescence in subcutaneous tumor experiment injected with or without 3 mg/kg Cu-CPT22. The yellow arrows indicate the positively stained cells. scale bars, 20 μm. (B) Representative images of immunostaining for Ki67 and CD31 in tumor tissue of subcutaneous tumor models with or without 3 mg/kg Cu-CPT22; black scale bars, 50 μm. (C) The positive ratio of CD31 in mice tumor tissue of subcutaneous tumor models. Data are shown as mean ± SD, ns: No statistical difference, **** P < 0.0001; ANOVA test (C).


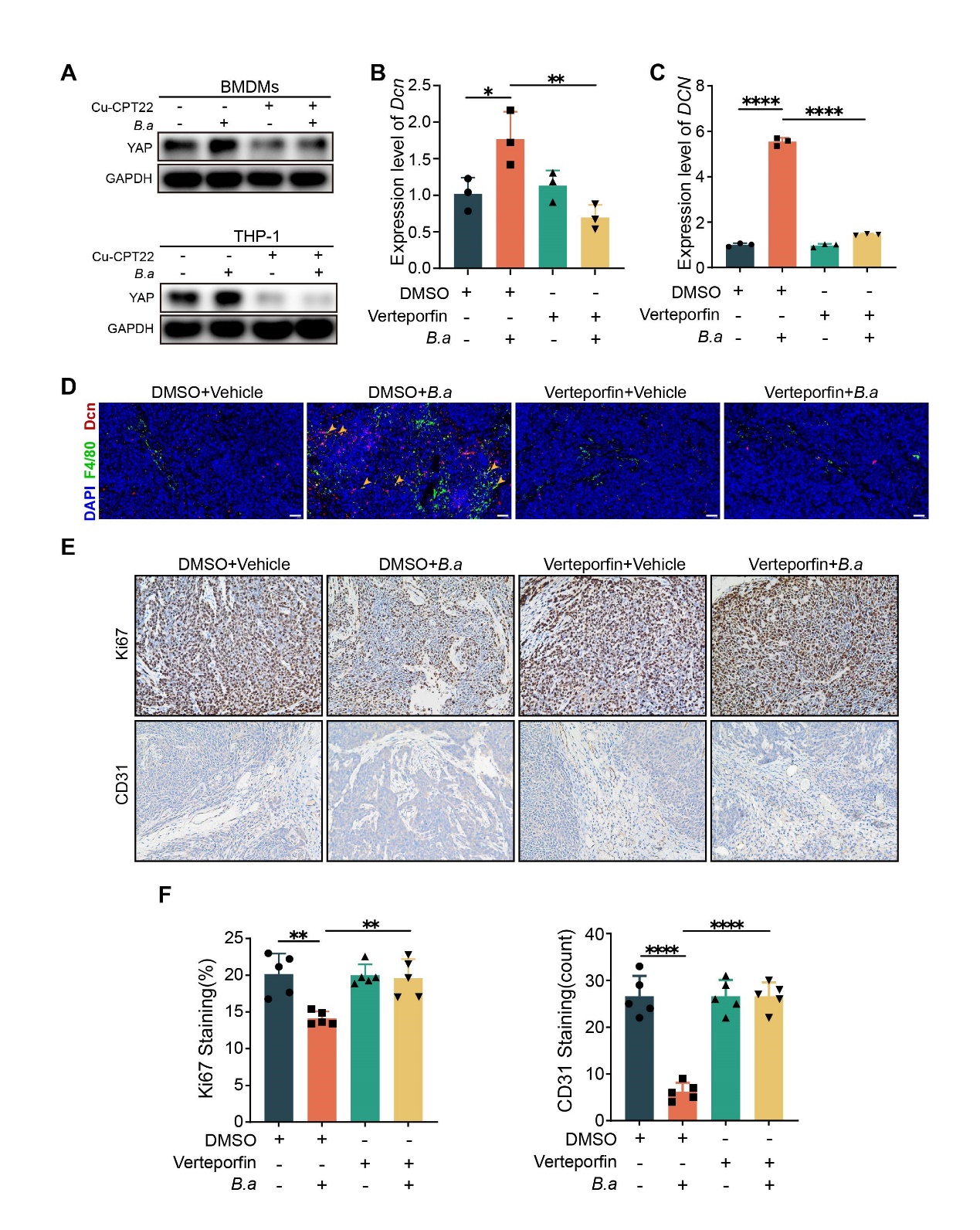


**Figure S6. *B.adolescentis* regulated DCN^+^ macrophages through TLR2/YAP axis.** (A) BMDMs and THP-1 cells were incubated with *B.adolescentis* or vehicle (PBS) for 24 hours with or without 25 μM Cu-CPT22. Protein level of YAP was tested by Western blot. (B-C) BMDMs (B) and THP-1 cells (C) cells were incubated with *B.adolescentis* or vehicle (PBS) for 24 hours with or without 1 μM verteporfin. mRNA level of *Dcn (DCN)* was tested by qRT-PCR; (D)The number of DCN^+^F4/80^+^ cells was examined by immunofluorescence in subcutaneous tumor experiment injected with or without 50 mg/kg verteporfin. The yellow arrows indicate the positively stained cells. scale bars, 20 μm. (E) Representative images of immunostaining for Ki67 and CD31 in tumor tissue of subcutaneous tumor models with or without 50 mg/kg verteporfin; black scale bars, 50 μm. (F) The positive ratio of Ki67 and CD31 in mice tumor tissue. The independent experiment was repeated three times. Data are shown as mean ± SD, * P < 0.05 ** P < 0.01 **** P < 0.0001, ANOVA test (B, C, F).


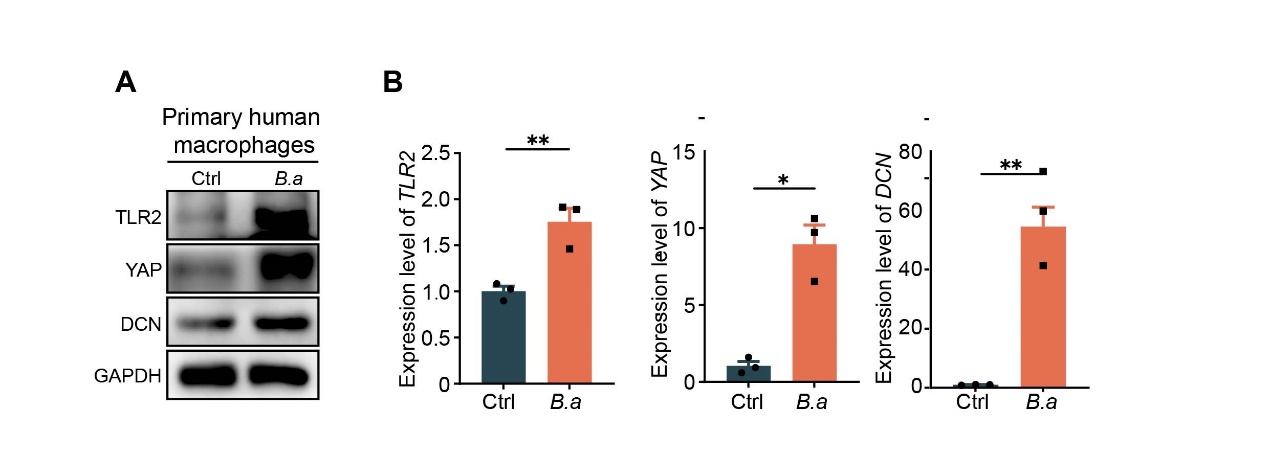


**Figure S7** ***B.adolescentis* activated TLR2/YAP/DCN in primary human macrophages.** (A-B) Primary human macrophages were incubated with *B.adolescentis* or vehicle (PBS) for 24 hours. The protein and mRNA levels of TLR2/YAP/DCN were tested in primary human macrophages. The independent experiment was repeated three times. Data are shown as mean ± SD, * P < 0.05 ** P < 0.01, Student *t* test (B).


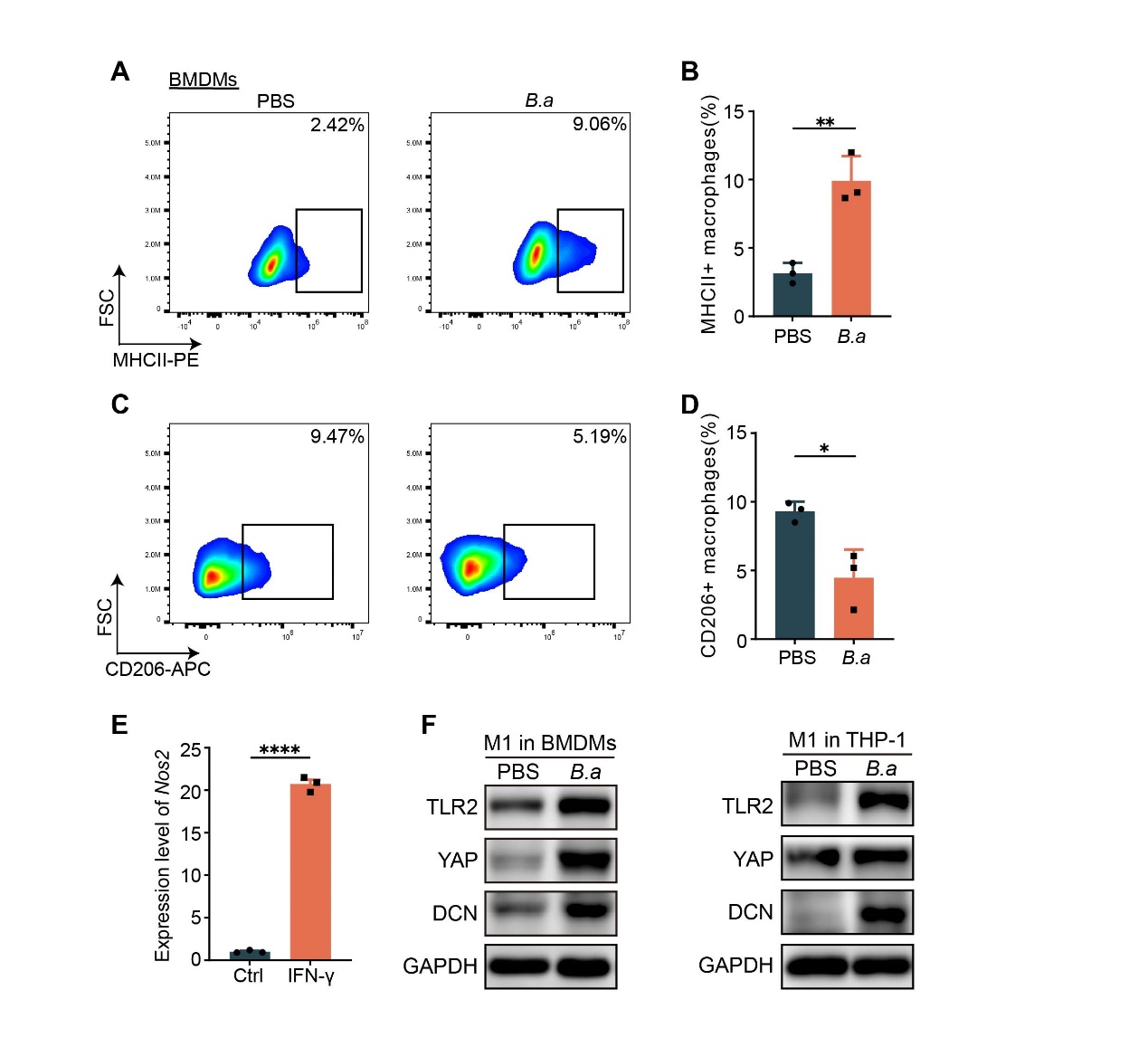


**Figure S8 *B.adolescentis* activated TLR2/YAP/DCN in M1 macrophages.** (A-D) Represent graphs and percentages of MHCII^+^ and CD206^+^ macrophages in *B.adolescentis-*treated BMDMs *in vitro* for 24 hours by flow cytometry. (E) The level of *Nos2* in BMDMs treated with IFN-γ (100 ng/mL) for 24 hours *in vitro* was tested by qRT-PCR. (F) BMDMs and THP-1 cells were stimulated with IFN-γ (100 ng/mL) for 24 hours and then incubated with *B.adolescentis* for 24 hours. Protein levels of TLR2, YAP and DCN were tested by Western blot. The independent experiment was repeated three times. Data are shown as mean ± SD, * P < 0.05 ** P < 0.01 **** P < 0.0001, Student *t* test (B, D, E).

**Supplementary Table S1: Primers used for validation the gene expression level**

| Primers | Sequencing(5ʹ–3ʹ) |
| --- | --- |
| *B.adolescentis -*F | *CTCCGCCGCTGATCCGGAAGTCG* |
| *B.adolescentis* -R | *AACCAACTCGGCGATGTGGACGACA* |
| *universal Eubacteria 16s-*F | *CGGCAACGAGCGCAACCC* |
| *universal Eubacteria 16s-*R | *CCATTGTAGCACGTGTGTAGCC* |
| Mouse *Gapdh-*F | *AACGACCCCTTCATTGAC* |
| Mouse *Gapdh-R* | *TCCACGACATACTCAGCA* |
| Mouse *Decorin-*F | *TCTTGGGCTGGACCATTTGAA* |
| Mouse *Decorin-R* | *CATCGGTAGGGGCACATAGA* |
| Mouse *Tlr2-*F | *GCAAACGCTGTTCTGCTCAG* |
| Mouse *Tlr2-R* | *AGGCGTCTCCCTCTATTGTATT* |
| Mouse *Tlr4-*F | *ATGGCATGGCTTACACCACC* |
| Mouse *Tlr4-R* | *GAGGCCAATTTTGTCTCCACA* |
| Mouse *Tlr5-*F | *GCAGGATCATGGCATGTCAAC* |
| Mouse *Tlr5-R* | *ATCTGGGTGAGGTTACAGCCT* |
| Mouse *Tlr9-*F | *ATGGTTCTCCGTCGAAGGACT* |
| Mouse *Tlr9-R* | *GAGGCTTCAGCTCACAGGG* |
| Mouse *Tlr13-*F | *GTTGTAACCTGGATGCCTAAGAC* |
| Mouse *Tlr13-R* | *GGCCTCTGTCAAGTTGGTGA* |
| Mouse *Sparc*-F | *TGGGAGAATTTGAGGACGGTG* |
| Mouse *Sparc*-R | *GAGTCGAAGGTCTTGTTGTCAT* |
| Mouse *Jund*-F | *GAAACGCCCTTCTATGGCGA* |
| Mouse *Jund*-R | *CAGCGCGTCTTTCTTCAGC* |
| Mouse *Col1a*-F | *GCTCCTCTTAGGGGCCACT* |
| Mouse *Col1a*-R | *ATTGGGGACCCTTAGGCCAT* |
| Mouse *Sqstm1*-F | *AGGATGGGGACTTGGTTGC* |
| Mouse *Sqstm1*-R | *TCACAGATCACATTGGGGTGC* |
| Mouse *Dusp1*-F | *GTTGTTGGATTGTCGCTCCTT* |
| Mouse *Dusp1*-R | *TTGGGCACGATATGCTCCAG* |
| Mouse M*arcksl1*-F | *CAATGGAGACTTAACCCCCAAG* |
| Mouse M*arcksl1*-R | *GGCCACTCAATTTGAAAGGCT* |
| Mouse *Junb*-F | *TCACGACGACTCTTACGCAG* |
| Mouse *Junb*-R | *CCTTGAGACCCCGATAGGGA* |
| Mouse *Ppp1r15a*-F | *GAGGGACGCCCACAACTTC* |
| Mouse *Ppp1r15a*-R | *TTACCAGAGACAGGGGTAGGT* |
| Mouse *Col3a1*-F | *CTGTAACATGGAAACTGGGGAAA* |
| Mouse *Col3a1*-R | *CCATAGCTGAACTGAAAACCACC* |
| Mouse *Hdc*-F | *CGTGAATACTACCGAGCTAGAGG* |
| Mouse *Hdc*-R  Mouse *Il6*-F  Mouse *Il6*-F  Mouse *Tnf*-F  Mouse *Tnf*-F | *ACTCGTTCAATGTCCCCAAAG*  *TAGTCCTTCCTACCCCAATTTCC*  *TTGGTCCTTAGCCACTCCTTC*  *CCCTCACACTCAGATCATCTTCT*  *GCTACGACGTGGGCTACAG* |
| Human *Β-ACTIN-*F | *AGAGCTACGAGCTGCCTGAC* |
| Human *Β-ACTIN-R* | *AGCACTGTGTTGGCGTACAG* |
| Human *Decorin-*F | *ATGAAGGCCACTATCATCCTCC* |
| Human *Decorin-R* | *GTCGCGGTCATCAGGAACTT* |
| Human *TLR2-*F | *GTTGCAAGCAGGATCCAAAGG* |
| Human *TLR2-R* | *TGAGCTGCCCTTGCAGATAC* |
